# The Transcriptional Coactivators p/CIP and SRC-1 Control Insulin Resistance through IRS1 in Obesity Models

**DOI:** 10.1371/journal.pone.0036961

**Published:** 2012-07-31

**Authors:** Zhiyong Wang, O. Jameel Shah, Tony Hunter

**Affiliations:** Molecular and Cell Biology Laboratory, Salk Institute for Biological Studies, La Jolla, California, United States of America; University of Bremen, Germany

## Abstract

Three p160 family members, p/CIP, SRC1, and TIF2, have been identified as transcriptional coactivators for nuclear hormone receptors and other transcription factors in vitro. In a previous study, we reported initial characterization of the obesity-resistant phenotypes of p/CIP and SRC-1 double knockout (DKO) mice, which exhibit increased energy expenditure, and suggested that nuclear hormone receptor target genes were involved in these phenotypes. In this study, we demonstrate that p/CIP and SRC1 control insulin signaling in a cell-autonomous manner both in vitro and in vivo. Genetic deletion of p/CIP and SRC-1 increases glucose uptake and enhances insulin sensitivity in both regular chow- and high fat diet-fed DKO mice despite increased food intake. Interestingly, we discover that loss of p/CIP and SRC-1 results in resistance to age-related obesity and glucose intolerance. We show that expression levels of a key insulin signaling component, insulin receptor substrate 1 (IRS1), are significantly increased in two cell lines representing fat and muscle lineages with p/CIP and SRC-1 deletions and in white adipose tissue and skeletal muscle of DKO mice; this may account for increased glucose metabolism and insulin sensitivity. This is the first evidence that the p160 coactivators control insulin signaling and glucose metabolism through IRS1. Therefore, our studies indicate that p/CIP and SRC-1 are potential therapeutic targets not only for obesity but also for diabetes.

## Introduction

The p160 coactivator proteins interact with and activate liganded nuclear hormone receptors, which function as transcription factors regulating target gene expression crucial for homeostasis, development, and reproduction [1]. These coactivators enhance transcriptional activities of most nuclear hormone receptors tested in vitro, including peroxisome proliferators-activated receptors (PPARs), with little specificity for individual hormone pathways [2], [3]. The PPAR family (including PPARα, β/δ, and γ) plays crucial roles in energy balance and glucose metabolism [1], [4]. Both in vitro and in vivo studies show that PPARγ is essential for adipogenesis, lipogenesis, and insulin sensitivity [1], [4]. PPARγ agonists such as thiazolidinediones (TZDs) have been used clinically as insulin sensitizers for treatment of diabetes, but with side effects including body weight gain and cardiac problems [1], [4]. Interestingly, partial loss of PPARγ in heterozygous knockout mice also increases insulin sensitivity in vivo [5], [6], [7], [8]. Whether and how the p160 coactivators play any direct role in glucose metabolism remain to be explored.

Mouse knockouts of individual p160 coactivators have distinct phenotypes: p/CIP (also called AIB1/ACTR/RAC3/NCoA1/SRC3) knockout mice have a somatic growth defect [9], [10], TIF2 (SRC2/GRIP2/NCoA2) knockouts are resistant to high fat diet-induced obesity with elevated adaptive thermogenesis [11], and SRC-1 knockouts exhibit an obese phenotype when challenged with a high fat diet [11], [12]. We have previously reported generation of p/CIP and SRC-1 double knockout mice (DKO), which have a lean phenotype despite higher food intake, in part due to an increased basal metabolic rate and elevated physical activity [13]. We showed that these coactivators function as adipogenic and lipogenic coactivators for PPARγ based on both in vitro differentiation assays of brown fat preadipocytes and gene deletion studies in vivo [13] The fascinating questions are what effect, if any, the coactivators exert on insulin signaling and glucose metabolism in vivo and whether the PPARγ pathway is involved.

In the current study, we examined the effects of loss of p/CIP and SRC-1 on insulin signaling and glucose metabolism, and discovered that p/CIP and SRC-1 negatively regulate the levels of IRS1 and insulin sensitivity both in vitro and in vivo. Our studies show that these coactivators play a crucial role in insulin resistance in obesity models in addition to their adipogenic and lipogenic activities. Our results demonstrate that reducing the levels and activities of p/CIP and SRC-1 results in increased insulin sensitivity and resistance to obesity. Therefore, p/CIP and SRC-1 are potential therapeutic targets for obesity and diabetes.

## Results

### p/CIP and SRC-1 DKO mice are more insulin sensitive

**Figure 1:**
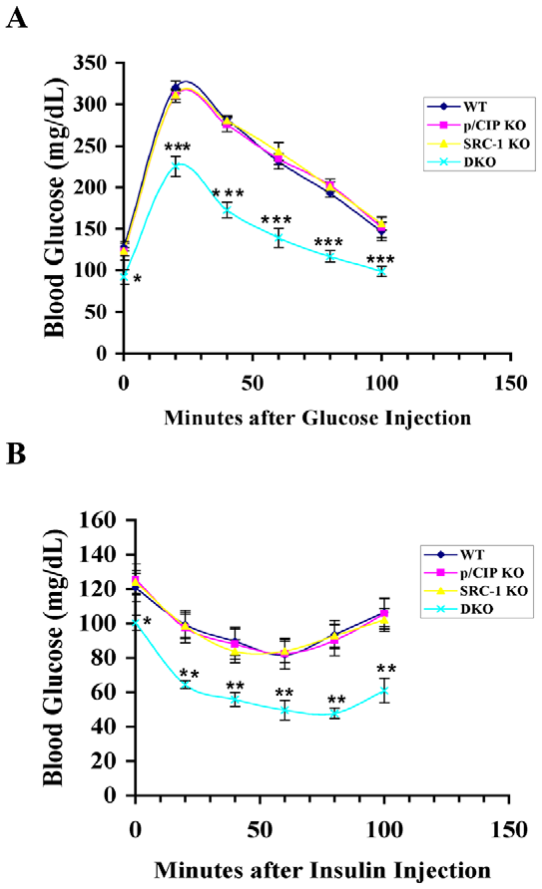
The DKO mice are more insulin sensitive in glucose and insulin tolerance tests (GTT and ITT respectively). A and B, Three-month old male mice (5 for each genotype) were fasted for six h, and intraperitoneally (ip) injected with a bolus of glucose (1.5 mg glucose/g body weight for GTT in panel A) or insulin (0.75 mU/g body weight for ITT in panel B). Blood glucose levels of mice were measured before and after injection with a glucometer at 20 min intervals. All results are presented as means ± S.E.M. * p<0.05, ** p<0.005, and *** p<0.0005.

In our initial report of the p/CIP and SRC-1 DKO mice, we observed a lean phenotype with regular chow, and resistance to obesity with a high fat diet [13]. To determine if the loss of p/CIP and SRC-1 affected glucose metabolism, we studied glucose uptake and insulin responses in three month old male mice on regular chow using standard glucose and insulin tolerance tests (Fig. 1A and B). We observed that the DKO mice had significantly lower fasting glucose levels, took up glucose much faster, and were significantly more insulin sensitive than their single knockout and wild-type littermates in both tolerance tests (Fig. 1A and B). There was no significant difference in either test between the single knockouts and wild-type littermates (Fig. 1A and B).

### p/CIP and SRC-1 DKO mice exhibit increased IRS1 levels and insulin signaling

**Figure 2:**
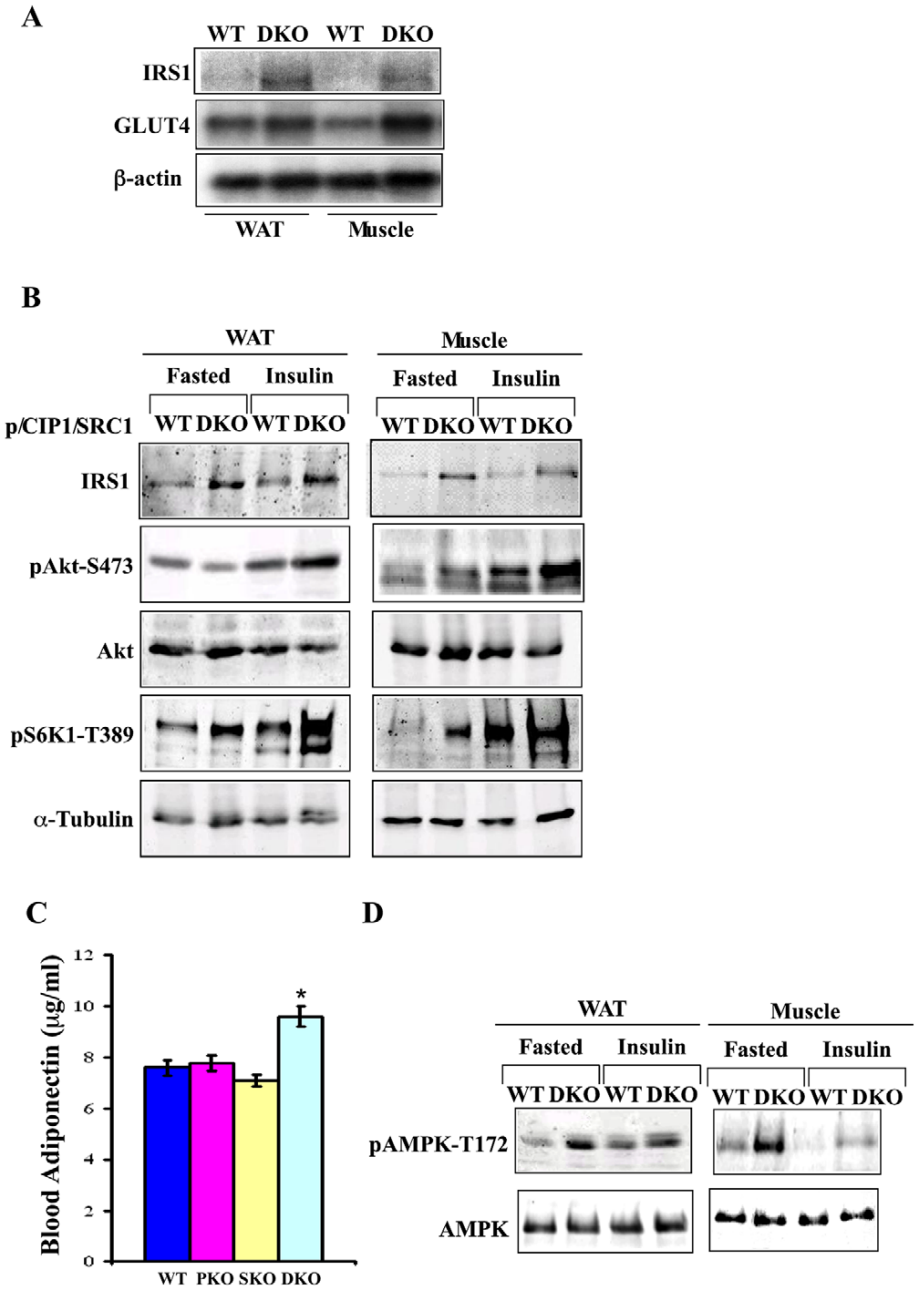
The DKO mice have increased IRS1 levels and insulin signaling. Three-month old mice were fasted overnight, injected (ip) either with insulin or with saline (fasted control), and mice were euthanized for tissue harvest. Cell lysates or total RNAs were prepared from white fat and muscle. A. Total RNAs isolated from white fat and muscle of fasted mice was used for Northern blot analyses. B. Tissue lysates of white fat and muscle of fasted and insulin treated mice were subjected to immunoblot analyses. C. DKO mice exhibited higher blood adiponectin levels. * p<0.05, ** p<0.005, and *** p<0.0005. D. Immunoblot analyses with AMPK and phospho-AMPK antibodies were performed.

To investigate the mechanism underlying the increased glucose uptake and insulin sensitivity, we studied expression levels of several components (such as insulin receptor, IRS1 and 2, AKT, GSK3, and S6 kinase) of the insulin pathway in DKO mice. The only significant change we observed was an increase in IRS1 RNA and protein levels in both white fat and muscle of DKO mice (Fig. 2A and B), where most of glucose uptake takes place. To determine if this was reflected in increased insulin pathway signaling, we fasted mice overnight, injected them with insulin intraperitoneally, harvested tissues 30 min later, and performed immunoblot analyses (Fig. 2B). We observed enhanced phosphorylation of AKT (Ser473) and S6K (Thr389) in fat and muscle (Fig. 2B) in DKO mice treated with insulin, compared to those of wild-type mice. Phosphorylation at Ser-473 activates the AKT kinase, which leads to phosphorylation of downstream targets such as GSK3. These results indicated that there was increased insulin signaling in fat and muscle of DKO mice, which are the two key tissues for glucose uptake and metabolism.

Adiponectin is produced and secreted from fat, and has been well established as an insulin-sensitizing adipokine [14]. We observed elevated blood levels of adiponectin in DKO mice (Fig. 2C), which correlated with increased mRNA levels of adiponectin in white fat (Wang and Hunter, data not shown). Adiponectin binds to its receptors and activates AMPK kinase [15], [16], [17], which leads to suppression of lipid and protein synthesis, and increased lipid oxidation and glucose uptake [18]. Consistent with elevated adiponectin, we observed increased AMPK phosphorylation on Thr172 (an activation marker) in the white fat and muscle of the DKO mice compared to that of wild-type littermates, both under fasting conditions and following insulin treatment for 30 min (Fig. 2D). We did not observe differences in AMPK phosphorylation between either of the single knockout mice and wild-type littermates (Wang and Hunter, data not shown). Insulin treatment suppressed AMPK activation in both tissues of wild-type and DKO mice as expected, but pThr172 AMPK levels remained higher in DKO than WT mice (Fig. 2D). Potentially, both increased blood adiponectin levels and elevated IRS1 levels in fat and muscle may enhance glucose metabolism and insulin sensitivity in the DKO mice.

### Knockdown of p/CIP and SRC-1 leads to increased IRS1 levels and insulin signaling

**Figure 3:**
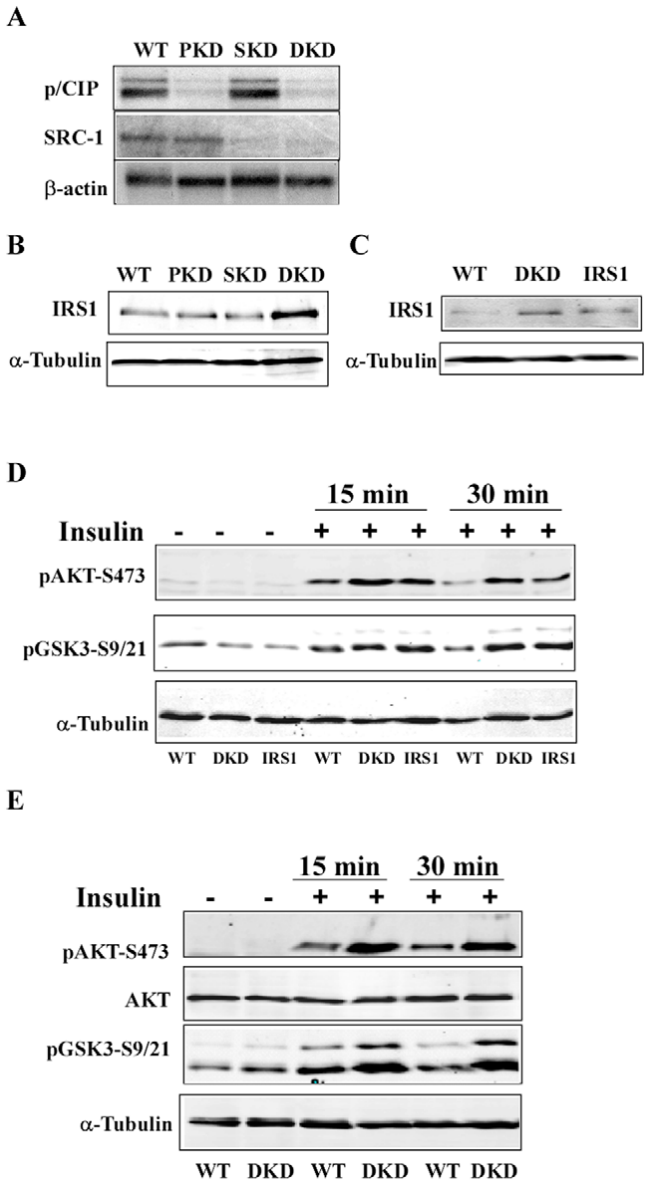
Knockdown of p/CIP and SRC-1 in two cell lines leads to increased IRS1 levels and enhanced insulin signaling. A. Lentiviral shRNA constructs of p/CIP, SRC-1, or both were used to stably knockdown endogenous levels of these coactivators in the F442A fibroblast cell line. 10 μg of total RNAs were used in Northern blot analyses to detect p/CIP and SRC-1 levels. A β-actin probe served as loading control. B. 50 μg total proteins of cell lysates from p/CIP knockdown (PKD), SRC-1 knockdown (SKD), wild-type, and double knockdown (DKD) were resolved by SDS-PAGE gel prior to immunoblot analyses. IRS1 protein levels were significantly increased in the DKD cell line, based on the α-tubulin loading control. C. Immunoblot analyses with stable p/CIP/SRC-1 DKD and IRS1 overexpression F442A cell lines. D. Serum starved F442A cell lines were stimulated for 15 or 30 min with 200 nM insulin, and immunoblot analyses were performed with the cell lysates using the indicated antibodies. Lanes 1, 4, and 7 were wild-type control with a vector construct only, lanes 2, 5, and 8 DKD cell line, and lanes 3, 6, and 9 IRS1 overexpression cell line. E. Immunoblot analyses with C2C12 myoblast stable DKD cell line. Lanes 1, 3, and 5 vector control, and lanes 2, 4, and 6 DKD.

To determine effects of these coactivators on insulin signaling in vitro, we used lentiviral based shRNA expression constructs to stably reduce endogenous p/CIP and SRC-1 levels in an NIH3T3 derived fibroblast cell line F442A, which is a preadipocyte cell line capable of differentiation into adipocytes in vitro. We obtained efficient knockdowns for p/CIP (PKD), SRC-1 (SKD), or both coactivators (double knockdown-DKD, Fig. 3A). We examined expression levels of target genes involved in insulin signaling and observed increased IRS1 mRNA (data not shown) and protein levels in the double knockdown (DKD) cell line but not in the single knockdowns (Fig. 3B). We examined acute insulin responses in the DKD cell line and observed increased phosphorylation of AKT (Ser473) and GSK3 (Ser9/21) with insulin stimulation (Fig. 3D), compared to vector-control F442A cells, whereas the insulin responses in single knockdown cells were not discernibly different from those control cells. These results demonstrate that loss of both p/CIP and SRC-1 increased IRS1 levels and enhanced insulin signaling in the F442A cell line.

To determine whether increased IRS1 levels were responsible for elevated insulin signaling, we established a stable F442A cell line overexpressing IRS1 at approximately the same level as in the DKD line (Fig. 3C). When stimulated with insulin, this stable cell line also exhibited similar increases in AKT and GSK3 phosphorylation. These results suggested that increased IRS1 levels in the DKD cell lines resulted in elevated cell-autonomous insulin signaling in vitro. To study these in vitro effects in a different cell line, we performed similar knockdown experiments in the C2C12 myoblast cell line, a representative muscle line. The combined knockdown of p/CIP and SRC-1 led to increased insulin signaling with elevated phosphorylation of AKT and its immediate downstream target GSK3 (Fig. 3E). Therefore, we demonstrated in two cell lines representing fat and muscle, the two key tissues for glucose uptake and metabolism in response to insulin, that loss of p/CIP and SRC-1 produced cell-autonomous increases of IRS1 and insulin signaling.

### p/CIP and SRC-1 DKO mice remain insulin sensitive despite high fat diet challenge

**Figure 4:**
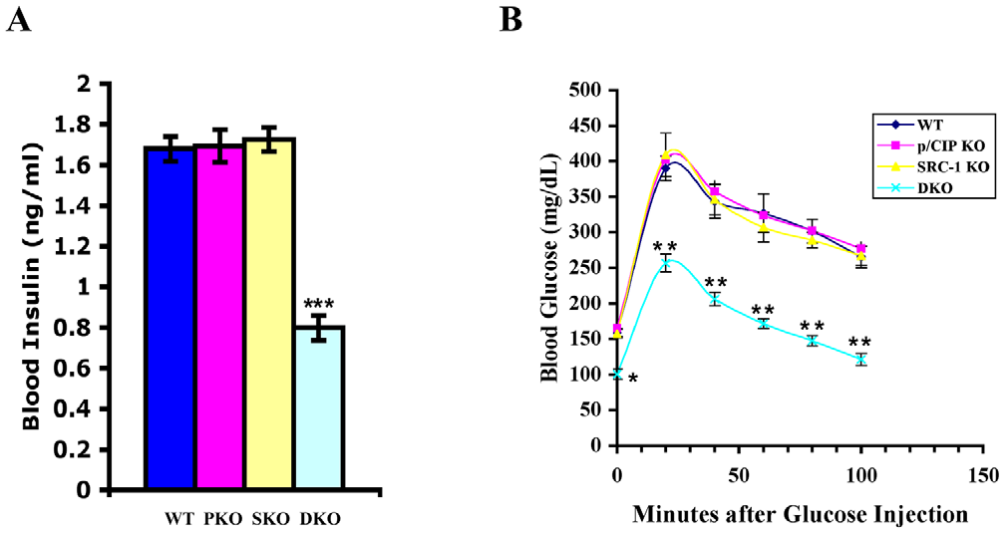
The DKO mice remain insulin sensitive on a high fat diet. A. After 10 weeks on the high fat diet, the male mice were fasted overnight, blood was drawn and sera were prepared for measurements of insulin with a radio-labeled immunoassay (5 each). The DKO mice had lower insulin levels with this diet than their littermate controls. B. The age-matched male mice were fasted for 6 h and glucose tolerance tests were performed after 10 week on the diet. Increased glucose uptake was observed in the DKO mice in GTT. * p<0.05, ** p<0.005, and *** p<0.0005.

The high fat diet common in developed countries results in not only obesity, but also insulin resistance and diabetes. To study the effects of loss of p/CIP and SRC-1 in a diet-induced obesity model, we challenged adult DKO mice (two months old) with a high fat diet (43% fat) for ten weeks. The wild-type and single knockouts gained 20–30% body weight on this diet, whereas the DKO mice gained considerably less weight despite significantly higher fat diet consumption [13]. However, these DKO mice also had significantly lower levels of total triglycerides, cholesterols, and fatty acids than those of single knockouts and wild-type littermates [13]. We observed that DKO mice had much lower fasting insulin levels than those of wild-type and single knockout mice with this diet (Fig. 4A). The DKO mice also exhibited much faster glucose uptake in a glucose tolerance test than their littermate controls on this diet (Fig. 4B). These results showed that the DKO mice were much more insulin sensitive despite higher food consumption with this high fat diet.

### The DKO mice are resistant to age-related obesity and glucose intolerance

**Figure 5:**
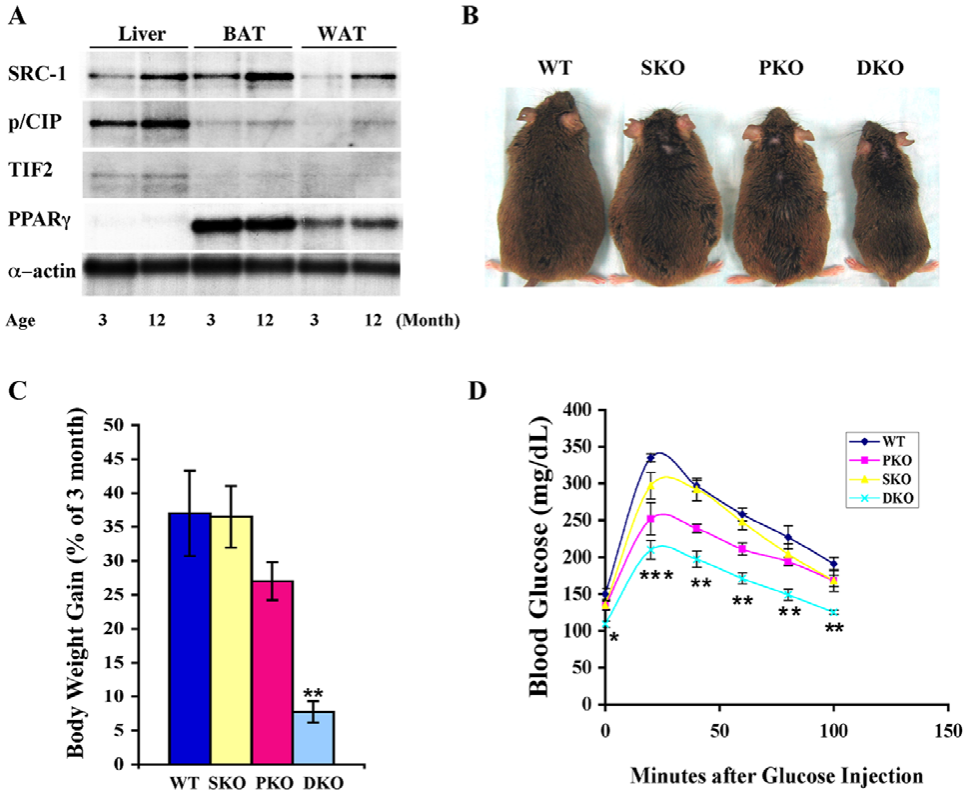
The DKO mice are resistant to age-related obesity and glucose intolerance. A. Northern blot analyses were performed to detect changes in p160 coactivator levels from 3 to 12 month old wild-type male mice. B. DKO mice remain lean at 12 months of age while the littermates become obese. C. Body weight gain is presented based on initial body weight of 3 month old male mice. D. GTT was performed with 12 month-old and sex-matched mice (5 for each genotype). * p<0.05, ** p<0.005, and *** p<0.0005.

We proceeded to investigate effects of p/CIP and SRC-1 on age-related obesity and glucose intolerance, and observed increased levels of p/CIP and SRC-1 in fat and liver of one year old wild-type male mice compared to those of 3 month old normal male mice (Fig. 5A). We did not observe any notable change in the levels of PPARγ, the master regulator of fat and glucose metabolism (Fig. 5A). Wild-type mice normally gain 30 to 40% body weight (based on 3 month body weights) for this period, most of which is fat (Fig. 5B and C). The increased expression of the p/CIP and SRC-1 coactivators correlated with the age-related body weight gain during this period (Fig. 5A and C). We then studied whether p/CIP and SRC-1 played a role in this type of obesity, and found that combined loss of these coactivators resulted in obesity resistant (Fig. 5B and C) and more glucose tolerant DKO mice at one year of age (Fig. 5D). Interestingly, we observed partial resistance to age-related obesity and glucose intolerance in the p/CIP single knockout mice (Fig. 5B–D). However, loss of both p/CIP and SRC-1 led to much greater resistance to this type of obesity and much better insulin responses, as was the case for DKO mice on high fat diet [13] (Fig. 4). Therefore, these results demonstrate that p/CIP and SRC-1 play redundant roles in controlling obesity and insulin resistance.

## Discussion

In the current study, we report that loss of the p/CIP and SRC-1 p160 coactivators increased insulin signaling and insulin sensitivity both in vitro and in vivo. We show that the effects are at least partially cell-autonomous, since both the preadipocyte F442A and myoblast C2C12 lines with double knockdowns of p/CIP and SRC-1 exhibited acutely increased insulin signaling. We demonstrated that the levels of IRS1 were elevated in the two knockdown cell lines, and that this change was responsible for increased insulin signaling in the F442A knockdown cell line, and potentially also in the DKO mice, which exhibit increased IRS1 mRNA and protein levels in white fat and muscle. We also showed that the DKO mice had increased glucose uptake and metabolism in high fat diet-induced and age-related obesity models. We believe that our discovery of the control exerted by the p160 transcriptional coactivators on IRS1 has significant implications for type 2 diabetes (T2D). Recent genome-wide association studies with human T2D patients have identified a genetic variant near the IRS1 gene associated with decreased IRS1 levels, increased T2D, insulin resistance and hyperinsulinemia [19]. These human studies correlate very well with mouse knockout studies of IRS1 [20], [21], [22], [23], and demonstrate key roles of IRS1 in insulin signaling and T2D. Our studies here show that p/CIP and αSRC-1 control IRS1 levels and determine insulin resistance in age-related and high fat diet-induced obesity models.

In our previous study, we reported that p/CIP and SRC-1 functioned as key coactivators for the PPAR γ pathway in adipogenesis and obesity, and these coactivators played redundant roles [13]. We observed the lean and obesity resistant phenotypes only in the DKO mice but not in either p/CIP or SRC-1 single knockout mice, because of increased basal metabolic rates and physical activity, which were similar to the phenotypes of PGC-1α knockout mice [13]. A recent study reported a lean and obesity-resistant phenotype in the p/CIP single knockout mice, which exhibited increased insulin sensitivity and elevated energy expenditure due to increased PGC-1α expression and its decreased acetylation by GCN5 [24]. However, they did not investigate the mechanism underlying increased insulin signaling in the p/CIP single knockout mice. In our current study, we did not observe a significant increase in insulin sensitivity in single p/CIP or SRC-1 knockout mice at three months of age, and found that only p/CIP/SRC-1 DKO mice exhibited insulin sensitive phenotypes on either regular chow or a high fat diet. Furthermore, we showed in this study that p/CIP and SRC-1 suppress IRS1 levels to control insulin resistance in different obesity models, which has not been reported before. The reason for the difference between the Coste et al. study [24] and our studies, in terms of obesity and insulin response upon loss of p/CIP in vivo, is currently unknown. Different genetic backgrounds and ages of mice used in the two studies may account for some of the differences. Interestingly, we did observe partial resistance to age-related obesity and glucose intolerance in p/CIP single knockouts (Fig. 5B and C), which was not observed in high fat diet-induced obesity models [13]. However, it is important to point out that even in the age-related obesity model, combined deletion of both p/CIP and SRC-1 resulted in much more drastic phenotypes in terms of obesity and insulin responses, illustrating again redundant roles of p/CIP and SRC-1 in this model. The difference between high fat diet-induced and age-related obesity of the p/CIP single knockout mice warrants further investigation.

In the p/CIP and SRC-1 DKO mice, some but not all the PPARγ target genes required p/CIP and SRC-1 for their expression [13]. PPARγ is crucial for insulin sensitivity and its agonists, such as TZDs, cause repartitioning of fat and lipids from insulin responsive tissues, such as muscle and liver, into white fat depots to enhance whole body insulin sensitivity; TZDs also function through PPARγ to regulate TNFα and resistin expression, both of which are involved in insulin resistance [1], [4]. Paradoxically, partial loss of PPARγ in heterozygous knockout mice also leads to increased insulin sensitivity and resistance to high fat diet-induced obesity because lower levels of PPAR γ result in decreases in overall body weight gains and fat contents 5,6,7. The phenotypes of the p/CIP and SRC-1 DKO mice are reminiscent of the lean and insulin-sensitive phenotypes of PPARγ heterozygotes. TZDs have been shown to reduce Ser307 phosphorylation of IRS1 by repressing production and secretion of TNFαand resistin from fat; this serine phosphorylation leads to impaired IRS1-mediated insulin signaling activity and IRS1 degradation [25], [26]. In the current study, we found that p/CIP and SRC-1 negatively regulate IRS1 mRNA and protein levels. Our results therefore uncouple the adipogenic, lipogenic, and obesity-inducing activities of p/CIP and SRC-1 as PPARγ coactivators from their activities on insulin signaling through IRS1. The mechanism underlying IRS1 regulation by these coactivators is unknown. We are actively investigating whether p/CIP and SRC-1 repress transcription of this key component of insulin signaling directly, or through induction of a repressor of IRS1 transcription.

We observed significantly higher levels of activated AMPK kinase in the DKO mice. We believe there are two reasons for that: one is that these mice have increased adiponectin levels, which may result from the lean phenotype of the DKO mice. Previous studies have established a correlation between lean subjects and increased adiponectin levels [27]. We have reported previously that the DKO mice are more active and have higher basal metabolic rates. Increased physical activity may also contribute to enhanced AMPK activity in the DKO mice [18]. AMPK activation results in extensive physiological changes, including decreased protein synthesis, higher levels of β-oxidation of fatty acids, and elevated expression of GLUT4 [18], which we also detect in the DKO mice (Wang and Hunter, data not shown). Together, these changes in insulin signaling and GLUT4 levels will facilitate faster and more efficient glucose uptake in the DKO mice.

In summary, our results suggest that targeting p/CIP and SRC-1 may not only reduce obesity but also enhance glucose uptake and insulin sensitivity, which would offer benefits not obtained by using TZDs for treatment of diabetes. We propose that decreasing the levels and/or activities of these coactivators may provide better solutions for obesity and diabetes in the future.

## Materials and Methods

### Animals

The homozygous knockout mice (DKO) were generated as described previously [13]. All animals were maintained with 12/12-light/dark cycles with free access to food and drinking water. A high fat diet (43% fat, TD97268) from Harland Teklad was fed to two-month old male littermate mice for ten weeks, and body weights were determined after ten weeks on the diet. All the animals were maintained and handled according to Institute and NIH Guidelines. All animal protocols and procedures were approved by the Salk Institute's IACUC Committee.

### Glucose and insulin tolerance tests and blood insulin and adiponectin measurements

Three-month old male mice were fasted for six h on the day of the experiment, and injected with a bolus of glucose (1.5 mg glucose/g body weight) or insulin (0.75 mU/g body weight) in PBS intraperitoneally. Blood glucose levels were measured with a glucometer (InDuo) and OneTouch Ultra strip from Lifescan, from tail bleeds before and after injection at 20 min intervals. To study insulin signaling in vivo, mice were fasted overnight and insulin was injected as above. After 30 min, mice were euthanized and tissues were dissected, harvested, and flash-frozen in liquid nitrogen. Blood insulin and adiponectin levels of overnight fasted, three-month old male mice were measured with radioimmunoassay kits from Linco.

### Tissue extraction and immunoblot analyses

White adipose tissue and muscle frozen in liquid nitrogen were extracted using a polytron tissue homogenizer in 7 volumes of ice-cold lysis buffer [40 mM HEPES, pH 7.5, 120 mM NaCl, 1% NP-40 (v/v) 1 mM EDTA, 10 mM pyrophosphate, 10 mM β-glycerophosphate, 50 mM NaF, 1 mM DTT, 1 mM Na_3_VO_4_, 1 mM PMSF, 1 μg/ml leupeptin, and 1 μg/ml aprotinin]. Extracts were solubilized on a vortex mixer for an additional 30 min at 4°C. Lysates were clarified by centrifugation at 13,000 ×g for 15 min at 4°C. Protein concentrations were determined spectrophotometrically using a BioRad DC protein assay kit. 30–50 μg of proteins were separated by SDS-PAGE and subjected to standard immunoblotting. AMPK, pAMPK-[T172], Akt, pAkt-[S473] and -[T308], pGSK-[S9/21], and pS6K1-[T389] antibodies were purchased from Cell Signaling Technology. A monoclonal antibody against α-tubulin was purchased from Sigma. The IRS1 antibody was from Santa Cruz Biotechnology.

### Northern blot analyses

Total RNAs from white fat and muscle were isolated with either TRIzol reagents (Invitrogen) or Qiagen's RNeasy Mini Kit, according to the manufacturers' protocols. Northern blot analyses were performed according to Current Protocols in Molecular Biology. Briefly, 10 μg RNA samples were denatured in formamide and formaldehyde and separated on a 1.2% denaturing agarose gel with 3% formaldehyde and 0.5 μg/ml ethidium bromide. Pictures of the agarose gel were then taken, and RNAs were transferred to Hybond-N+ nylon membrane with 20x SSC by blotting overnight. Denatured 32P-labeled cDNA probes were then hybridized with the membranes in Stratagene's Quik-Hyb solution at 65°C for 1 h. The membranes were washed once with 2× SSC and 0.1% SDS, once with 0.5× SSC and 0.1% SDS, each at room temperature for 15 min, and then 0.1% SSC and 0.1% SDS at 65°C for 15 min. Membranes were exposed to X-ray film at −80°C overnight with a screen. After stripping of signals with boiling 0.5% SDS, subsequent hybridizations were performed on the same membranes with indicated probes.

### Knockdown of endogenous p/CIP and SRC-1 in F442A and C2C12 cell lines

293T and C2C12 cell lines were from ATCC, and F442A was a generous gift from Dr. Peter Tontonoz [28]. Four shRNA targeting sequences each for p/CIP and SRC-1 were chosen from the RNAi Consortium of the Broad Institute. Synthetic DNA oligonucleotides were cloned into the pLKO1 lentiviral vector and lentivirus stocks were produced in 293T cells by transfections with shRNA producing plasmids. The viruses were used to infect F442A and C2C12 cells and stable cell lines were obtained by selecting with puromycin (final concentration of 400 μg/ml) for two weeks. The surviving clones were pooled for each virus and targeting efficiencies were tested by Northern blotting. The most efficient knockdown cell lines were chosen for subsequent experiments for single knockdowns, or for double knockdown with another round of infections. The chosen shRNA sequences were: p/CIP: 5′-AATTCAAAAA**CGGCAGGCACTTGAAATGAAA**CTCGAG**TTTCATTTCAAGTGCCTGCCG** ; SRC-1: 5′- AATTCAAAAA**CAGCAGCTACTGACTGAATAAC**TCGA**GTTATTCAGTCAGTAGCTGCTG** **.** The high-lighted sequences targeted p/CIP or SRC-1 respectively. The knockdown cell lines were used to examine levels of components of insulin signaling pathway including IRS1. To investigate insulin response, the cells were serum-starved for 24 h, and 200 nM insulin was added for 15 or 30 min. Cells were harvested to make lysates for immunoblot analyses.

### Statistical analyses

All results are presented as means ± S.E.M. A non-paired student *t* test was used for these analyses. A difference is considered significant as the following: * p<0.05, ** p<0.005, and *** p<0.0005.
